# Anatomical variations of coronary venous system and its tributaries: A cadaveric study

**DOI:** 10.1186/1749-8090-10-S1-A255

**Published:** 2015-12-16

**Authors:** Suhani Sumalatha, Vrinda Hari, Lydia S Quadros, Antony S D'Souza

**Affiliations:** 1 Department of Anatomy, Kasturba Medical College, Manipal University, Manipal, 576108, India

## Background/Introduction

Knowledge of the coronary venous system (CVS) anatomy is an important factor before many electrophysiological procedures, such as CRT or ablations.

## Aims/Objectives

The aim of the present study is to note the anatomy of the coronary venous system and its tributaries in cadaveric hearts.

## Method

Total of fifty-five formalin-fixed adult human cadaveric hearts studied. Following measurements were noted: a.) length of coronary sinus, b). its relation to left coronary artery, mitral valve annulus and left atrium, c.) number of atrial and ventricular tributaries, d.) distance and the opening angles of major tributaries from the coronary ostium, e.) length and width of coronary ostium, f.) Attachment of Thebesian valve.

## Results

The following results are obtained: a) Length of coronary sinus (CS) ranged from 2 cm to 3.8 cm, the mean being 2.8 cm. b) Relation of coronary sinus to the left coronary artery (LCA) and mitral valve annulus was above and parallel in 100% cases c) The number of Atrial tributaries ranged from 1-2 and ventricular from 1-6. d) The mean distance of Anterior interventricular vein (AIV), Posterior vein of the left ventricle (PVLV), Oblique vein of left atrium (OVLA), Middle cardiac vein (MCV) from the coronary ostium was 67.5 mm, 32 mm, 41 mm, 7 mm respectively e) The average length and width of coronary ostium was 9 mm and 13 mm respectively. f) the besian valve in 24/55 hearts was attached to the superior, right and inferior margins of the ostium. In 29/55 hearts to the inferior margin of the ostium.

## Discussion/Conclusion

For invasive cardiologists, knowledge about CVS anatomy could add value before and during electrophysiology procedures.

